# Heterogeneous Expression of PD-L1, B7x, B7-H3, and HHLA2 in Pulmonary Sarcomatoid Carcinoma and the Related Regulatory Signaling Pathways

**DOI:** 10.3390/cancers15133372

**Published:** 2023-06-27

**Authors:** Feng Wang, Ayse Ece Cali Daylan, Lei Deng, Jihua Yang, Janaki Sharma, Christopher Su, Shenduo Li, Xingxing Zang, Balazs Halmos, Alain Borczuk, Haiying Cheng

**Affiliations:** 1 Department of Oncology, Albert Einstein College of Medicine, Bronx, NY 10461, USA; 2 Department of Oncology, Montefiore Medical Center, Bronx, NY 10467, USAbahalmos@montefiore.org (B.H.); 3 Department of Medicine, Roswell Park Comprehensive Cancer Center, Buffalo, NY 14203, USA; 4 Department of Medicine, University of Miami Health System, Miami, FL 33136, USA; 5 Department of Medicine, University of Washington, Seattle, WA 98195, USA; 6 Department of Medicine, Mayo Clinic Comprehensive Cancer Center, Jacksonville, FL 32224, USA; li.shenduo@mayo.edu; 7 Department of Pathology, Weill Cornell Medicine, New York, NY 10065, USA

**Keywords:** immune checkpoint, pulmonary sarcomatoid sarcoma, *MET*ex14, PD-L1, signaling pathways

## Abstract

**Simple Summary:**

Pulmonary sarcomatoid carcinoma (PSC) is an aggressive subtype of non-small-cell lung cancer (NSCLC). It does not respond favorably to standard chemotherapy, and the response to PD-1/PD-L1 inhibitors remains modest. The introduction of new therapeutic approaches for this subtype is crucial. Our study demonstrates that PD-L1 expression was significantly higher in the epithelial component than in the sarcomatoid component. Expression of PD-L1 in both components was only seen in 32.1% of patients. However, the majority of PSC patients had at least one immune checkpoint expression in both components. Thus, combination immune checkpoint inhibition based on expression profiles may prove as a personalized and effective treatment strategy. This study also reveals a high rate of *MET* exon 14 skipping mutation (*MET*ex14) in PSC. *MET*ex14 selectively induced PD-L1 expression through MAPK or PI3K/Akt pathways. A combination of targeted therapies with immunotherapy in this population also warrants further investigation as a novel treatment approach.

**Abstract:**

Immunotherapy has transformed lung cancer management, but PSC remains an aggressive subtype with a poor prognosis. This study investigates the differential expression of PD-L1 and alternative immune checkpoints (ICs; B7x, B7-H3, and HHLA2), and genetic alterations in PSCs. Tumor specimens of 41 PSC patients were evaluated. PD-L1, B7x, B7-H3, and HHLA2 were positive in 75.0%, 67.6%, 73.0%, and 91.9% of tumors, respectively. PD-L1 expression was significantly higher in the epithelial compared to the sarcomatoid component (median TPS: 50% vs. 0%, *p* = 0.010). Expression of PD-L1 in both components was only seen in 32.1% of patients. However, at least one IC was expressed in 92.9% of epithelial and 100% of sarcomatoid components. Furthermore, *MET*ex14 was detected in 19.5% of patients and was associated with a higher sarcomatoid percentage. Our preclinical studies revealed that *MET*ex14 induced PD-L1 expression via MAPK or PI3K/Akt pathways, and MET inhibitors decreased PD-L1 expression. Our findings demonstrate distinct expressions of ICs in PSC subcomponents. Thus, combination IC inhibition as a therapeutic strategy in PSC warrants further exploration. A high percentage of *MET*ex14 in PSC and its role in regulating PD-L1 expression reveal different therapeutic targets in this aggressive NSCLC subtype.

## 1. Introduction

PSC is a rare and aggressive subtype of NSCLC. It accounts for about 0.5% of NSCLC cases diagnosed in the United States and is associated with poor survival rates [1,2]. PSC is a heterogeneous entity encompassing a broad histological spectrum of disease, subcategorized into pleomorphic carcinoma, spindle cell carcinoma, giant cell carcinoma, carcinosarcoma, and pulmonary blastoma [3].

The NSCLC treatment paradigm and clinical outcomes have shifted drastically with the incorporation of targeted therapy and immunotherapy in the last several decades. Despite these advancements, PSC remains a subtype with a poor prognosis necessitating further investigation of underlying oncogenic pathways, which may ultimately lead to new biomarker-driven therapeutic approaches. In particular, the heterogeneous nature of PSC with varying levels of epithelial and sarcomatoid components requires better characterizing the differing immune checkpoint expression and actionable genetic alterations in these distinct compartments, which may offer a more personalized therapeutic approach for these patients.

PSC has been demonstrated to respond poorly to first-line platinum-based chemotherapy [4]. It is highly aggressive and has worse overall survival compared to the other subtypes of NSCLC [5,6]. Hence, the evaluation of novel therapies in PSC is crucial. A better understanding of immune evasion pathways in PSC is fundamental to maximizing the benefits of immunotherapy. PD-L1 expression is reported to be higher in PSC than in conventional NSCLC, leading to the hypothesis of increased immunotherapy efficacy in this subgroup. Multiple retrospective studies demonstrated an overall response rate of 40–55% upon treatment with PD-1/PD-L1 inhibitors in the PSC population [7,8]. In addition, the use of immunotherapy has produced durable responses in rare PSC patients [9]. Nonetheless, despite a high level of PD-L1 expression in PSC, these response rates to PD-1/PD-L1 inhibitors remain modest. Therefore, extending durable immunotherapy benefits to a broader population in PSC is critical. One promising strategy for achieving this is combination immune checkpoint inhibition as a therapeutic approach. In addition to the PD-L1 and CTLA-4 axis, several additional potentially actionable immune checkpoint molecules have recently been discovered, including the group III molecules of the B7-CD28 immune checkpoint family, which are B7x (B7-H4/B7S1), B7-H3 (CD276), and HHLA2 (B7H7/B7-H5/B7y) [10]. These alternate immune checkpoints predominantly act as co-inhibitors of T-cell function but also may have other functions depending on the engagement of different receptors or immune microenvironment [11,12,13]. Understanding the immune evasion pathways of these molecules, especially in NSCLC, is crucial since they are reported to be commonly expressed in 37–69% of this subgroup of solid tumors [14,15,16,17]. Effective inhibition of these alternative immune checkpoints may prove a promising therapeutic strategy, especially in PD-L1 negative NSCLC, which has been shown to express these alternative molecules widely or in association with primary resistance to PD-1/PD-L1 blockade [14]. As the understanding of these alternate immune checkpoints improves and potent inhibitors are developed, preclinical studies for some are now advancing into clinical trials [10,18].

Sarcomatoid carcinomas are hypothesized to evolve from a shared precursor through epithelial–mesenchymal transition (EMT) [19]. Phylogenetic and genomic profiling of two components of PSC demonstrated that the sarcomatous component arises from an epithelial precursor early in the course of cancer evolution and accumulates different genetic alterations [20]. Similarly, another study investigating gene signatures in biphasic PSCs also revealed that loss of the epithelial-associated transcription factor promotes the development of the sarcomatoid phenotype by expressing EMT-driving transcription factors [21]. As these sarcomatoid carcinomas seem to arise and differentiate from their precursor cells, understanding and therapeutically targeting both components are essential to improve clinical outcomes. In fact, the HGF (hepatocyte growth factor)/MET pathway has been hypothesized to affect the motility and differentiation of epithelial cells and play a role in epithelial–mesenchymal interaction [22,23]. Therefore, genetic alterations of the *MET* gene and their contribution to the pathogenesis of PSC require further elucidation. In fact, studies of genetic alterations in PSC revealed a high percentage of tumor protein p53 (*TP53*) (74%), KRAS proto-oncogene, GTP-ase (*KRAS*) (34%), MET proto-oncogene receptor protein kinase (*MET*) (13.6%), and epidermal growth factor receptor (*EGFR*) (8.8%) variants [24]. Our group also reported enrichment of *MET*ex14 in PSC patients, occurring in about 20% compared to 0.5–3% in other NSCLC subtypes [25,26,27]. Interestingly, similar to the PSC population, *MET*ex14 mutant NSCLC has also demonstrated high levels of PD-L1 expression [28,29]. Whole-transcriptome sequencing of a large cohort of *MET*ex14 also revealed a highly immunosuppressive tumor microenvironment with a wide expression of immune checkpoints and inflammatory signature due to IFN-γ signaling [29]. Considering these findings, a more detailed understanding of the regulatory mechanisms of immune checkpoints in *MET*ex14 variants is crucial for biomarker-driven therapeutic approaches.

This study investigates the heterogeneity of PD-L1 and alternative immune checkpoints B7x, B7-H3, and HHLA2 in differing PSC components harboring genetic alterations of *MET*, *KRAS*, *EGFR*, and *ALK*. In addition, given early evidence of enrichment of *MET*ex14 mutation in PSC and high levels of PD-L1 expression, this study also aims to explore cellular pathways that may explain this association.

## 2. Materials and Methods

### 2.1. Patients

Forty-one patients with pathologically confirmed PSC were identified from a previously reported clinical cohort of patients with NSCLC. The clinicopathological data were collected through retrospective chart review, and the tissue microarray method was implemented, as described previously [14]. All protocols were reviewed and approved by the Institutional Review Board.

### 2.2. Immunohistochemistry

The immunohistochemistry (IHC) staining method used in this study was derived from prior studies [14,15,30]. In brief, tumor tissues were fixed in 10% neutral-buffered formalin at room temperature for 24 h. The formalin fixed lung tumor tissue was baked at 60 °C for 1 h to overnight followed by deparaffinization and rehydration of tumor tissue sections using xylene and ethanol. The slides were immersed in Dako dual endogenous enzyme block for 10 min at room temperature to block endogenous peroxidase activity (Dako corporation, S2003). Dako target retrieval citrate solution pH 6.1 (Agilent Dako code number S1699) was used for the antigen retrieval step. The slides were heated in the microwave at 900 W for 2 min, placed in a steamer for 30 min, and left to cool for 20 min at room temperature. Dako Protein Block Serum-Free (Agilent Dako code number X0909) was used to block nonspecific binding. The primary antibodies used in this study and incubation details are available in Table 1.

Dako Envision system-HRP labeled polymer anti-mouse system (Dako Corporation code K4000) was used and incubated for 30 min at room temperature, followed by DAB chromogen staining (Vector laboratories SK-4100) and hematoxylin nuclear counterstaining. PD-L1, B7x, HHLA2, and B7-H3 expression was evaluated by two independent investigators and quantified as 0, 1, 2, and 3 for absent, low, moderate, and high expression. The H-score was calculated by multiplying the percentage of staining (proportion score) measured by Image J and an ordinal value corresponding to the maximum intensity score in the specimen. PD-L1 staining was reported as a tumor proportion score (TPS).

Tumor-infiltrating lymphocyte (TIL) scores were read by visual estimation of the proportion of lymphocytic infiltration in each histospot of the same hematoxylin- and eosin-stained slides, as previously described [15].

### 2.3. Cell Culture

H292 and H125 cell lines were purchased from the American Type Culture Collection (Manassas, VA, USA). H292 and H125 cells were maintained in RPMI-1640 medium and supplemented with 10% FBS fetal bovine serum (FBS) with penicillin and streptomycin (Life Technologies, Carlsbad, CA, USA) at 37 °C under 5% CO_2_.

### 2.4. CRISPR

As previously described, two guide RNAs (gRNAs) separately targeting *MET* Exon 13 and Exon 15 were designed using the CRISPR design tool (http://crispr.mit.edu/, accessed on 1 February 2017) to generate *MET*ex14 [34]. gRNA1 (5′-CTTGTTAAAGACGGCTATCA-3′) and gRNA2 (5′-ACCCACTGAGGTATATGTAT-3′) were cloned into the BbsI site of plasmid PX458 (SpCas9-2A-EGFP) (Addgene: #48138). The transfection step was completed using Lipofectamine 3000 (Life Technologies, Carlsbad, CA, USA), and single-cell clones were selected using flow cytometry.

### 2.5. RNA Sequencing

Following incubation in a serum-free medium for 18 h, the cells were treated with HGF (100 ng/mL) for 0, 6, and 24 h. The cells were collected, and total RNA was extracted using an RNeasy mini kit (QIAGEN, Valencia, CA, USA). RNA-seq libraries were prepared with the TruSeq sample preparation kit (Illumina, San Diego, CA, USA). The sequencing was performed using the BGI2000 platform with paired-end reads of 200 bp. We sequenced 18 samples in total and the reads in all the samples satisfied the quality control by FastQC (v0.11.9) [35]. The reads were mapped to the human reference genome (GRCh38) using STAR aligner (v2.6.1b) [36], and alignments were guided by a human gene annotation (Gencode primary assembly annotation v30). More than 96% of reads in individual samples were successfully and uniquely mapped, and the mismatch rate was less than 0.35%. We employed HTSeq (v0.6.1) [37] to count reads from the output (bam files) of the STAR aligner and identified >17,700 expressed genes per sample. We performed the standard protocols of STAR and HTSeq in this study, with the default parameters. The raw counts of samples were normalized on the basis of their library sizes, and the differential gene expression analyses were performed on the basis of the negative binomial distribution using the ‘DESeq2’ package (v1.26.0) [38].

### 2.6. Quantitative PCR

Quantitative reverse transcription PCR (qRT–PCR) assays were performed to measure the mRNA expression of multiple immune checkpoints. Total RNA was extracted from treated cells using an RNeasy Mini Kit (QIAGEN, CA). cDNA was synthesized from 1 μg of purified total RNA with the SuperScriptTM IV First-Strand cDNA synthesis system using oligo(dT) primers (Life Technologies, Carlsbad, CA, USA), and qPCR was performed using CFX96 Real-Time PCR Detection System with the following primer sequences (Table 2).

All gene expression levels were normalized to the reference gene expression, and relative changes in expression were calculated by the 2^−ΔΔCT^ formula.

### 2.7. Flow Cytometry

For PD-L1, B7-H3, and B7x expression, cells pretreated at the indicated conditions were stained with APC anti-hPD-L1 (clone 29E.2A3), PE/Cy7 anti-hB7-H3 (clone MIH42), PE anti-hB7x (clone MIH43), or isotype controls for 30 min at 4 °C. Fluorophore-conjugated antibodies were purchased from BioLegend. The staining of HHLA2 was performed by first incubating cells with primary mouse anti-hHHLA2 mAb (clone B5B5) [13] or control mouse IgG1 (clone MOPC-21) for 40 min at 4 °C, followed by staining with APC polyclonal goat F(ab′)2 anti-mouse IgG Fc (eBioscience) for 30 min at 4 °C. Cells were washed after staining and analyzed on an LSR II (BD Biosciences, San Jose, CA, USA). Data were analyzed with FlowJo (FlowJo, LLC, Ashland, OR, USA).

### 2.8. Western Blot

Total proteins were extracted from treated cells with RIPA buffer (50 mM Tris-HCl pH 7.5, 150 mM NaCl, 1% NP-40, 0.5% sodium deoxycholate, 0.1% SDS, and 1 mM EDTA) supplemented with the protease and phosphatase inhibitor cocktail (Thermo Fisher Scientific, Rockford, IL, USA). The cell lysate was separated by SDS polyacrylamide gel electrophoresis (SDS-PAGE) and transferred to the nitrocellulose membrane (Millipore, St. Louis, MO, USA). The membranes were soaked with 5% skimmed milk (in PBS) for 1 h and then incubated with the primary antibodies (Table 3) at 4 °C overnight.

The membranes were incubated with a horseradish peroxidase (HRP)-conjugated secondary antibody (Cell signaling technology catalog no 7074, Danvers, MA, USA) at 1:2000 dilution for 1 h, and the image was developed with an ECL detecting kit (Amersham Biosciences, Piscataway, NJ, USA, catalog no RPN2108).

### 2.9. Statistical Analysis

The χ^2^ test or Fisher exact test was used to compare the distribution of each categorical variable. The Wilcoxon rank-sum test was used for the nonparametric comparison of distribution. Logistic regression was used to adjust variables for multivariate analyses. Survival analysis was performed by log-rank test and further adjusted by Cox regression. All tests were completed as two-sided using IBM SPSS Statistics (version 22).

Statistical analysis for in vitro cell-based experiments was performed using GraphPad Prism (version 8.0). The two-sided *t*-test was used to estimate the statistical significance of differences between the two groups. Two-way ANOVA with Bonferroni correction was used to determine statistical significance for real-time quantitative PCR analysis. Data are presented as the mean ± standard deviation.

Statistical significance is indicated as follows: * *p* < 0.05, ** *p* < 0.01, and *** *p* < 0.001. All *p*-values ≤0.05 were considered statistically significant.

## 3. Results

### 3.1. Clinicopathological Characteristics, Immune Checkpoint Expression, and Genetic Alterations in PSC Patients

The cohort’s median age was 73 years (range: 38–87). Gender distribution was similar with 48.8% of the patients (20/41) being male. Of the patients with known smoking status (*n* = 38), 7.3% were never smokers. Among the cohort, 29.3%, 41.5%, 24.4%, and 4.9% of patients had stage I, II, III, and IV disease, respectively. Among patients with available subtype information (*n* = 37), 97.3% had pleomorphic carcinoma, and 2.7% had spindle cell carcinoma. The epithelial histology was the most dominant component across the PSC samples with a median of 70% per sample (interquartile range: 26–85%), followed by a sarcomatoid component of 30% (interquartile range: 15–50%) and spindle cell component of 25% (interquartile range: 15–50%).

**Figure 1:**
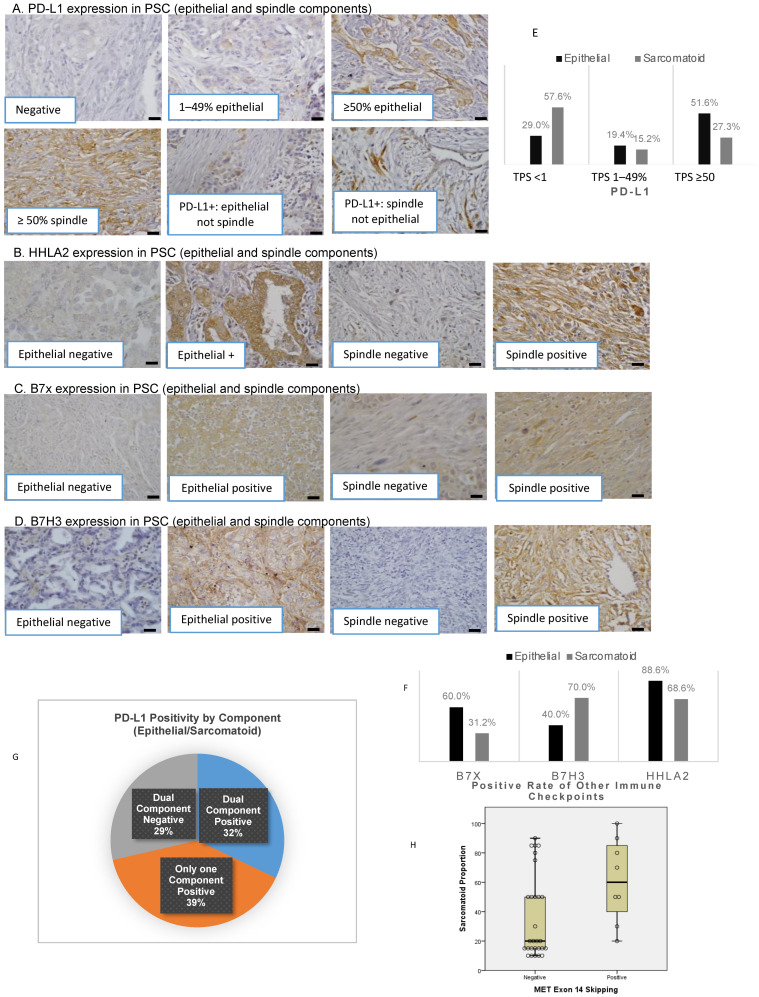
Immune checkpoint distribution and the correlation of sarcomatoid percentage with genetic alterations. Immunohistochemical staining of PD-L1 (**A**), HHLA2 (**B**), B7x (**C**), and B7-H3 (**D**) in PSC scale bar 20 µm. Distribution of PD-L1 (**E**) and alternate immune checkpoint molecules (**F**) by histologic components. The distribution of PD-L1 positivity in histologic subcomponents (**G**). The correlation of sarcomatoid percentage with *MET*ex14 (**H**).

*MET*ex14 and *KRAS* mutations were found in 19.5% and 14.6%, respectively, while *EGFR/ALK* alterations were absent. PD-L1, B7x, B7-H3, and HHLA2 were positive in 75.0% (27/36), 67.6% (25/37), 73.0% (27/37), and 91.9% (34/37) of patients, respectively (representative images in Figure 1). A TIL proportion equal to or greater than 30% was found in 28.6% (10/35) patients. There were no significant associations between clinicopathological factors and the expression of any of the four immune checkpoints (Table 4).

### 3.2. Distinct Expression of Immune Checkpoints in Epithelial and Sarcomatoid Components

PD-L1 expression was significantly higher in the epithelial compared to the sarcomatoid component (median TPS: 50% vs. 0%, *p* = 0.010, H-score 75 vs. 0, *p* = 0.014, Table 5A). The percentage of TPS <1%, 1–49%, and ≥50% differed between epithelial and sarcomatoid components (29.0% vs. 57.6%, 19.4% vs. 15.2%, and 51.6% vs. 27.3%, Figure 1E). Analogous to PD-L1, B7x expression was more enriched in the epithelial than in the sarcomatoid component (TPS 25 vs. 0, *p* = 0.008). In contrast, there was a trend toward higher expression of B7-H3 in the sarcomatoid component (TPS 0% vs. 50%, *p* = 0.053, H-score epithelial 0% vs. 75%, *p* = 0.176, Table 5A). There was no significant difference found in HHLA2 expression between the two components.

Although PD-L1 was positive in most tumors, dual expression of PD-L1 in epithelial and sarcomatoid components was only present in 32.1% (9/28) of patients (Figure 1G). PD-L1 was expressed in only one component in 39% of patients, and PD-L1 was negative in both components in 29% of patients.

PD-L1 expression was not associated with the other three immune checkpoints (Table 4). At least one immune checkpoint was expressed in 92.9% of epithelial and 100% of sarcomatoid components (Table 5B). In tumors with negative PD-L1 in both components, HHLA2 was expressed in 100% of epithelial and 66.7% of sarcomatoid components (Table 5C). In tumors with negative PD-L1 in both components and negative HHLA2 in the sarcomatoid component, B7-H3 was 100% positive in the sarcomatoid component.

### 3.3. Association of Epithelial and Sarcomatoid Distribution in PSC Samples with Oncogenic Genomic Alterations and Immune Checkpoints

Tumors harboring *MET*ex14 had significantly higher sarcomatoid percentage (median 60%, interquartile range: 35–88% vs. *MET* wildtype 20%, interquartile range: 15–50%, *p* = 0.024, Figure 1H). After adjusting for age, smoking, stage, and gender, a trend toward a higher sarcomatoid percentage in this subpopulation was preserved (*p* = 0.055). There was no significant association between higher sarcomatoid percentage with *KRAS* mutation or positivity of immune checkpoints.

### 3.4. Genomic Alterations and Their Association with Immune Checkpoints and Tumor-Infiltrating Lymphocytes

*MET*ex14 and *KRAS* mutations were mutually exclusive in our cohort. *MET*ex14 was associated with a higher B7-H3 H-score in the sarcomatoid component compared to the wildtype (*p* = 0.017, Supplementary Table S1). *MET*ex14 was also associated with a higher HHLA2 H-score in both epithelial (*p* < 0.001) and sarcomatoid (*p* = 0.031) components (Supplementary Table S1). *MET*ex14 tumors also had numerically higher PD-L1 TPS in the sarcomatoid component compared to *MET* wildtype (*p* = 0.079). *KRAS* mutation was significantly associated with a lower HHLA2 H-score in the epithelial component (*p* = 0.041).

PSC samples with *MET*ex14 were more likely to have higher tumor-infiltrating lymphocytes (*p* = 0.027, Supplementary Table S2). However, after adjusting for age, gender, smoking, and stage, the association was no longer significant (*p* = 0.093). There were no significant associations between *KRAS* mutation and tumor-infiltrating lymphocytes (Supplementary Table S2).

### 3.5. Survival Analysis

The median survival of the entire cohort was 1020 days (95% CI: 643–1397). After adjusting for age, gender, smoking, stage, genetic mutation, and expression of immune checkpoints, a higher percentage of the sarcomatoid component was associated with shorter survival (continuous variable, HR 1.048, 95% CI: 1.007–1.080). After adjusting for age, gender, smoking, and stage, the lack of B7-H3 expression in the sarcomatoid component was significantly associated with better survival (HR 0.034, 95% CI 0.002–0.741). However, there was no significant association between B7-H3 expression in the epithelial component and patient survival (Supplementary Table S3).

### 3.6. HGF/METex14 Signaling Specifically Induces PD-L1 Expression

**Figure 2:**
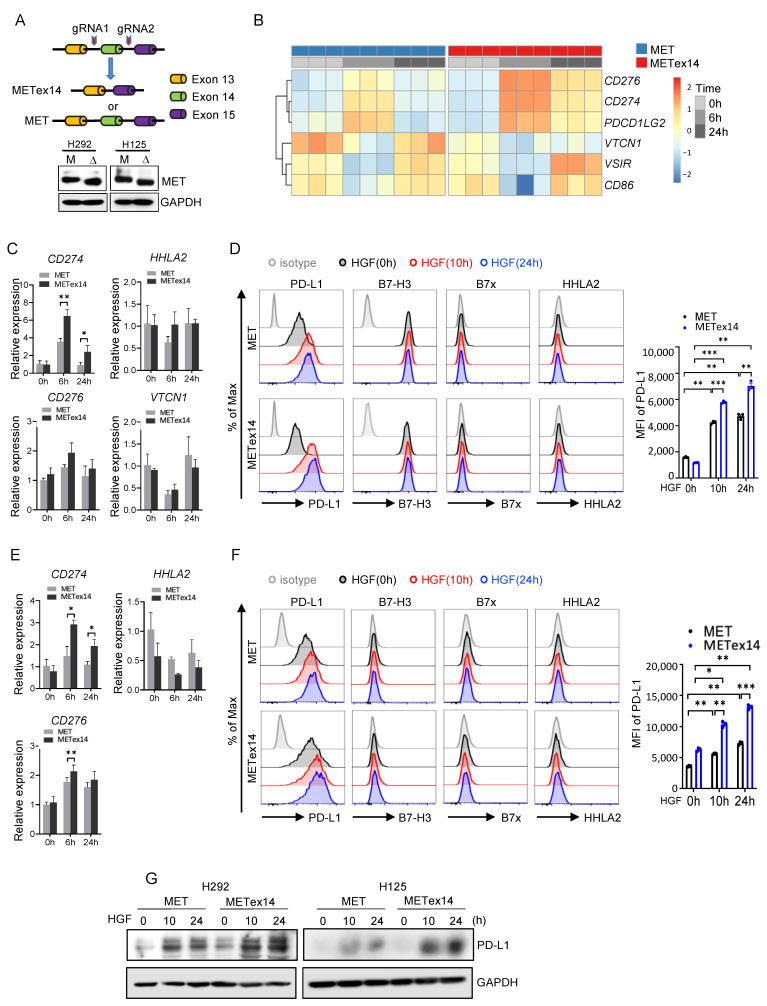
Effect of HGF/*MET*ex14 signaling on expression of multiple immune checkpoints. (**A**) Schematic diagrams of the establishment of *MET*ex14 cell models using CRISPR gene editing technology. Western blot analysis of *MET* WT and *MET*ex14 expression in H292 and H125 cells. M: MET; Δ: *MET*ex14. (**B**) RNA sequencing analysis was pursued on H292 *MET* WT and *MET*ex14 cells treated with HGF at indicated times. The expression profile of a group of immune checkpoints is shown. (**C**–**F**) Analysis of expression level of multiple immune checkpoints in *MET* WT and *MET*ex14 cells treated with HGF at indicated timepoints. The fold changes of *CD276*, *CD274*, *VTCN1*, and *HHLA2* mRNA expression using qRT-PCR (H292 cells in **C**, H125 cells in **E**), and flow cytometry analysis of expression of PD-L1, B7-H3, B7x, and HHLA2 (H292 cells in **D**, H125 cells in **F**) are shown. (**G**) Western blot analysis of PD-L1 expression in *MET* WT and *MET*ex14 cells exposed to HGF at indicated times. MFI: mean fluorescence intensity. The two-sided *t*-test was used to estimate the statistical significance of differences between the two groups. Two-way ANOVA with Bonferroni correction was used to determine statistical significance for real-time quantitative PCR analysis. * *p* < 0.05; ** *p* < 0.01; *** *p* < 0.001. The uncropped blots are shown in File S1.

*MET*ex14 lung cancer cell line models were established using CRISPR technology, where deletion of *MET* exon 14 in both alleles was regarded as mutated (*MET*ex14), and cell lines with intact *MET* exon 14 were used as wildtype (Figure 2A). RNA-sequencing analysis was pursued to clarify the global transcriptome signatures underlying HGF/*MET*ex14 signaling. As shown in Figure 2B, the expressions of *CD276* (*B7-H3*), *CD274* (*PD-L1*), and programmed cell death 1 ligand 2 (*PDCD1LG2*, *PD-L2*) were enhanced post 6 h and 24 h HGF treatment. In contrast, the expressions of V-Set immunoregulatory receptor (*VSIR*, *VISTA*), *CD86*, and V-Set domain containing T-cell activation inhibitor 1 (*VTCN1*) genes were decreased at 6 h and reversed to a baseline level at 24 h post HGF treatment in both *MET*ex14 and *MET* WT cell lines (Figure 2B). qRT-PCR and flow cytometry analyses also confirmed these observations. As shown in Figure 2C, the expression level of *CD274* mRNA was markedly increased at 6 h followed by a decrease at 24 h of HGF treatment in both *MET* and *MET*ex14 cell lines. In addition, *CD274* mRNA expression levels were significantly higher in both 6 h and 24 h in *MET*ex14 compared to *MET* cells. However, mRNA expression of *CD276*, *VTCN1*, and *HHLA2* did not show a significant change in exposure to HGF in *MET* WT and *MET*ex14 cells (Figure 2C). Simultaneously, flow cytometry analyses validated that HGF treatment dramatically enhanced the PD-L1 and B7-H3 expression at all timepoints over 24 h but did not affect B7x and HHLA2 expression in either H292 *MET* WT or *MET*ex14 cell lines. The comparison between *MET*ex14 and *MET* WT cell lines revealed that HGF treatment led to significantly higher PD-L1 but similar B7-H3 expression in *MET*ex14 cells (Figure 2D). Similar results were obtained in modified H125 cells except for the lack of increase in B7-H3 by HGF treatment (Figure 2E,F). Furthermore, we also validated that HGF/*MET*ex14 signaling promotes PD-L1 expression using a Western blot assay in a time-dependent manner (Figure 2G). These results suggest that HGF/*MET*ex14 signaling significantly increases PD-L1 expression in both cell lines, whereas it might not affect B7-H3, B7x, and HHLA2 expression.

### 3.7. METex14 Drives PD-L1 Expression via PI3K/Akt and MAPK Signaling Cascades, and MET Inhibitors Block PD-L1 Expression

**Figure 3:**
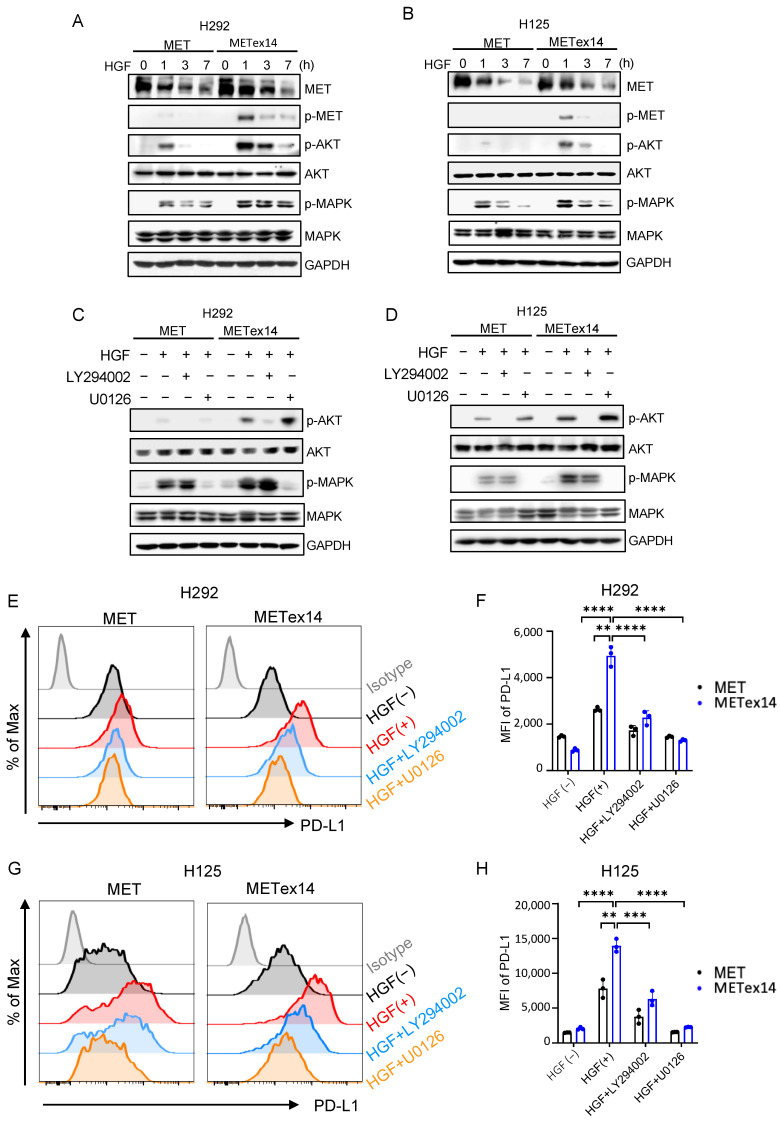
HGF/*MET*ex14 signaling significantly drives the expression of PD-L1 via activation of PI3K/Akt and MAPK pathways. (**A**,**B**) Effect of HGF/*MET*ex14-mediated downstream signaling. *MET* WT and *MET*ex14 cells (H292 (**A**) and H125 (**B**)) treated with HGF (100 ng/mL) at indicated timepoints were harvested, and phosphorylated and total protein levels of MET, Akt, and MAPK were measured using Western blot. (**C**–**H**) Blockade of PI3K/Akt and MAPK pathways inhibited HGF/*MET*ex14-triggered PD-L1 expression. *MET* WT and *MET*ex14 cells (H292 (**C**) and H125 (**D**)) treated with HGF with or without LY294002 (PI3K inhibitor, 20 μM) or U0126 (MAPK inhibitor, 10 μM) for 3 h were harvested. Western blot analysis of phosphorylated and total protein levels of Akt, MAPK, and GAPDH are shown. Flow cytometry analysis of PD-L1 expression in *MET* and *MET*ex14 cells in exposure to HGF at indicated times (H292 (**E**,**F**) and H125 (**G**,**H**)) are shown. MFI: mean fluorescence intensity. The two-sided *t*-test was used to estimate the statistical significance of differences between the two groups. Two-way ANOVA with Bonferroni correction was used to determine statistical significance for real-time quantitative PCR analysis. ** *p* < 0.01; *** *p* < 0.001; **** *p* < 0.0001. The uncropped blots are shown in File S1.

To understand how HGF/*MET*ex14 signaling regulates PD-L1 expression, we investigated the PI3K/Akt and MAPK signaling cascades. Similar to our prior work, HGF treatment delayed receptor degradation and significantly increased the activity of PI3K/Akt and MAPK in the *MET*ex14 cell line compared to the *MET* WT cell line (Figure 3A,B) [34]. We next assessed the effect of LY294002 (PI3K inhibitor) and U0126 (MAPK inhibitor) on HGF-dependent PD-L1 expression. As expected, LY294002 and U0126 markedly repressed HGF-driven p-Akt and p-MAPK in both *MET* and *MET*ex14 cell lines (Figure 3C,D). More importantly, both LY294002 and U0126 dramatically inhibited HGF-elicited PD-L1 expression in both *MET* and *MET*ex14 cell lines (Figure 3E,F in H292 cells, and Figure 3G,H in H125 cells). These results indicated that HGF/*MET*ex14 signaling significantly increases PD-L1 expression via PI3K/Akt and MAPK pathways.

**Figure 4:**
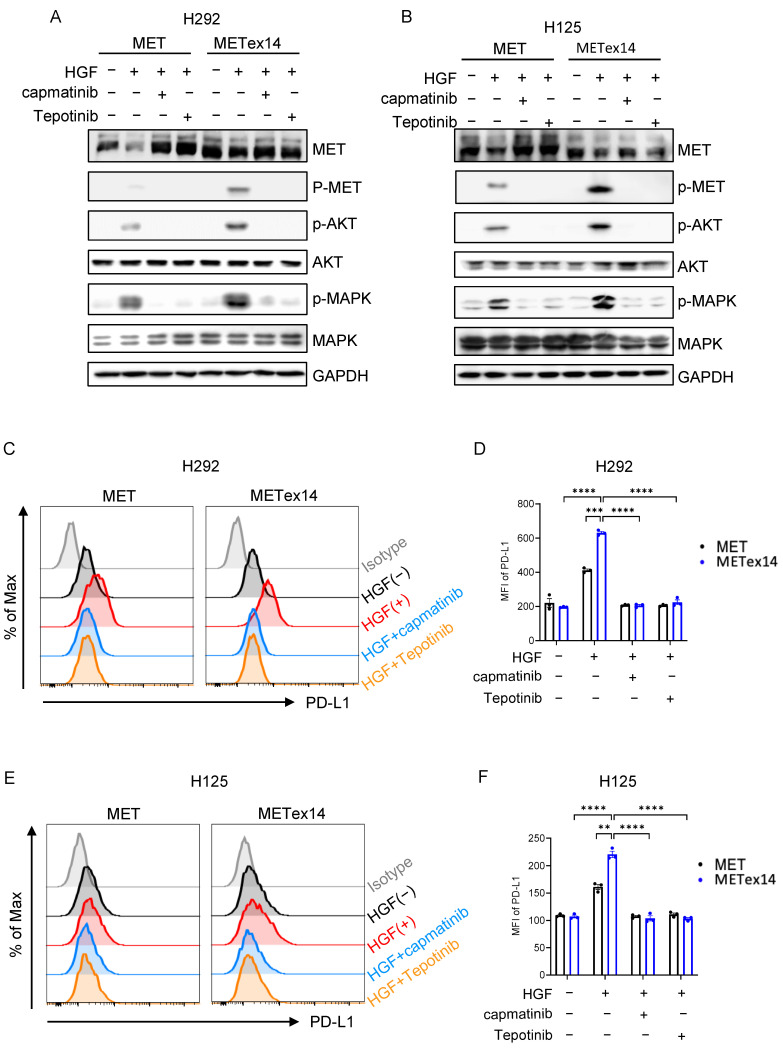
Effect of MET inhibitors, capmatinib and tepotinib, on HGF/*MET*ex14-triggered PD-L1 expression. (**A,B**) Effect of MET inhibitors, tepotinib or capmatinib, on *MET*ex14-mediated receptor degradation and downstream signaling. *MET* WT and *MET*ex14 cells treated with HGF (100 ng/mL) with or without capmatinib (100 nM) or tepotinib (100 nM) for 1 h were lysed, and phosphorylated and total protein levels of MET, AKT, and MAPK were measured using Western blot (H292 (**A**) and H125 (**B**)). (**C**–**F**) The effect of capmatinib or tepotinib on HGF/*MET*ex14-triggered PD-L1 expression. Flow cytometry analysis of PD-L1 expression in *MET* and *MET*ex14 cells in exposure to HGF with capmatinib or tepotinib at indicated times (H292 (**C**,**D**) and H125 (**E**,**F**)) are shown. MFI: mean fluorescence intensity. The two-sided *t*-test was used to estimate the statistical significance of differences between the two groups. Two-way ANOVA with Bonferroni correction was used to determine statistical significance for real-time quantitative PCR analysis. ** *p* < 0.01; *** *p* < 0.001; **** *p* < 0.0001. The uncropped blots are shown in File S1.

These results raise the possibility that inhibition of MET signaling might repress PD-L1 expression. We next assessed this possibility using two MET inhibitors: capmatinib and tepotinib. Capmatinib, similar to our prior work [34], and tepotinib markedly repressed MET receptor phosphorylation and downstream Akt and MAPK phosphorylation (Figure 4A,B). More importantly, capmatinib and tepotinib treatment vigorously suppressed HGF-induced PD-L1 expression in both *MET* and *MET*ex14 cell lines (Figure 4C,D in H292 cells, Figure 4E,F in H125 cells). These results demonstrate that MET inhibitor reduces HGF/*MET*ex14 signaling-driven PD-L1 expression in both H292 and H125 NSCLC cell lines.

## 4. Discussion

Given the known heterogeneity of PSC, our study investigated the differential expression of immune checkpoints in epithelial and sarcomatoid components, as well as their associations with genetic alterations. Prior studies have reported high PD-L1 expression in the PSC population, consistent with our study results [39,40]. However, the reports of the distribution of PD-L1 expression in epithelial and sarcomatoid components of PSC are few and demonstrated variable findings [20,41]. Our study is the first to report that PD-L1 expression is more enriched in the epithelial compared to the sarcomatoid component. These differences may stem from different patient characteristics or antibodies.

In our cohort, dual component positivity of PD-L1 was only seen in 32% of tumors. While the intricate mechanism of differential PD-L1 expression in these two components currently remains unclear, it may result in important therapeutic consequences. For example, despite higher PD-L1 TPS in PSC, the heterogeneity of PD-L1 expression in the various components may limit the efficacy of single-agent immunotherapy. Our group previously showed that the majority of PD-L1 negative NSCLC expresses alternative immune checkpoints, such as B7x and HHLA2 [14]. In addition, recently, HHLA2’s interaction with KIR3DL3 (killer cell Ig-like receptor, three Ig domains, and long cytoplasmic tail 3) was demonstrated to inhibit the function of CD8+ T and NK cells, and a first-in-class monoclonal antibody, NPX267, targeting KIR3DL3 enhanced antitumor immunity in HHLA2^+^ tumors [13,42]. In this PSC cohort, over 90% of PSC tumors expressed at least one immune checkpoint (B7x, B7-H3, HHLA2, or PD-L1) in both components. Of note, HHLA2 was almost universally present across the whole cohort, with positive rates above 90%. A pooled analysis of 90 PSC patients treated with single-agent PD-1/PD-L1 inhibitors revealed an overall response rate (partial or complete response) of 54.5% even though 74.2% of patients had PD-L1 TPS ≥ 50% [7]. Thus, despite high PD-L1 expression, increasing the response rates in this aggressive tumor type remains a challenge. According to our study findings, treating PSC patients with combination immune checkpoint inhibitors regimens tailored based on detailed histopathologic reporting of component-specific immune checkpoint expressions may overcome immunotherapy resistance and increase treatment efficacy.

In addition to delineating the heterogeneity of PSC, our study demonstrates that these subcomponents and immune checkpoint expression levels may affect clinical outcomes. A higher sarcomatoid percentage is associated with worse survival, independent of age, gender, stage, genetic mutation, and expression of immune checkpoints. In addition, the lack of B7-H3 in the sarcomatoid component was independently associated with better survival.

These findings argue for more detailed pathological reporting of PSC, including the percentages of subcomponents due to potential correlations with survival and varying immune checkpoint molecule expression that may guide therapeutic management. Furthermore, reporting PD-L1 TPS across the whole specimen could overestimate the potential benefit of PD-1/PD-L1 monotherapy since the sarcomatoid component may express a disproportionately low PD-L1 level. As prospective data regarding the biomarker value of B7x, B7-H3, and HHLA2 molecules and the efficacy of their inhibitors accumulate, pathology reports of PSC may need to include the immune checkpoint expression levels by histologic components.

Our PSC cohort was enriched in *MET*ex14 variants (19.5%), followed by *KRAS* mutations (14.6%). In contrast to other studies reporting *ALK* and *EGFR* alterations in PSC, we did not find any *ALK*/*EGFR* alterations, possibly due to the higher smoking rate and lesser frequency of Asian patients in our cohort [24,27]. In addition, patients with *MET*ex14 had a numerically higher sarcomatoid percentage, which could be secondary to the role of the HGF/*MET*ex14 pathway in epithelial–mesenchymal transition and evolution of sarcomatoid components in PSC (Figure 1H). Interestingly, the *MET*ex14 variant also showed higher PD-L1 expression, yet the underlying cellular pathways remain elusive. Using a pair of *MET* WT and *MET*ex14 lung cancer cell lines models, we demonstrated that the HGF/*MET* signaling pathway plays a critical role in enhancing PD-L1 expression with higher levels of PD-L1 in *MET*ex14 cell lines compared to *MET* WT. Of note, by using CRISPR technology to develop *MET*ex14 cell line models, our study enables investigation of the specific effect of *MET*ex14 alteration independent from that of *MET* amplification. Intriguingly, our results highlight the role of *MET*ex14 signaling in selectively increasing the expression of the immune checkpoint, i.e., PD-L1 expression, compared to the other immune checkpoints, B7x, B7-H3, and HHLA2. The low baseline expression of these alternative immune checkpoints in parental cells and the regulation of expression in an HGF/*MET*ex14-independent pathway may explain these findings.

In this study, HGF/*MET*ex14 signaling significantly increased the activation of downstream PI3K/Akt and MAPK pathways. Moreover, inhibition of PI3K and MAPK pathway markedly repressed HGF-dependent PD-L1 expression, suggesting that either PI3K/Akt or MAPK cascades are tightly involved in the upregulation of PD-L1 expression. These results may partially elucidate the mechanism underlying high PD-L1 expression in *MET*ex14 variants and, therefore, could offer a rationale for combination therapy strategies consisting of PD-L1 and MAPK or PI3K/Akt pathway inhibition as a treatment strategy in this subpopulation. Multiple clinical trials investigating the effect and safety of MAPK pathway inhibitors with PD-L1/PD-1 inhibitors in NSCLC are currently ongoing (NCT03581487, NCT03600701). Subgroup analyses for *MET*ex14 patients in these trials should be pursued as these patients may demonstrate better clinical outcomes with combination therapy.

MET-inhibiting TKIs, such as capmatinib and tepotinib, have shown promising results in clinical trials for NSCLC patients with *MET*ex14 or *MET* amplification, leading to accelerated FDA approvals [43,44]. In our study, both capmatinib and tepotinib rigorously repressed *MET*ex14-mediated PD-L1 expression. These results revealed that MET inhibitors at least partially inhibit PD-1/PD-L1 signaling. However, despite high PD-L1 expression in *MET*ex14 NSCLC, the efficacy of PD-1/PD-L1 inhibitors seems lower than expected, with reported overall response rates ranging between 16% and 43% [28,45,46]. On the basis of the demonstration of the dynamic nature of PD-L1 expression with MET inhibitors demonstrated in our study, the timing and sequence of targeted and immunotherapy warrant further investigation.

Our study is limited by the small sample size; however, this is expected given the low prevalence of PSC as a rare subtype of NSCLC. Yet another limitation is the retrospective nature of our study. Although we separately reported the IHC of two components, tumors were not microdissected for further genetic studies.

## 5. Conclusions

Heterogeneity of immune checkpoint expression exists between epithelial and sarcomatoid components in PSCs, with only one-third of PSCs expressing PD-L1 in both components. Alternate immune checkpoints are also widely expressed in PSCs, and further studies exploring the use of combinations of new immune checkpoints in this population are necessary. Furthermore, given the association of *MET*ex14 and higher sarcomatoid percentage, further studies investigating the underlying mechanism of sarcomatoid differentiation in this subpopulation are warranted. Our study also sheds light on the mechanisms underlying the upregulation of PD-L1 by HGF/*MET*ex14 signaling via MAPK and PI3K/Akt pathways. In addition, by demonstrating the dynamic nature of PD-L1 levels via MET inhibition in the *MET*ex14 variant, future investigators may be better poised to explore new targeted and immunotherapy combinations and optimize their timing.

## Figures and Tables

**Table 1:** Antibodies used for immunohistochemistry.

Protein	Clonality	Clone	Company	Catalog No.	Dilution and Incubation Time
PD-L1	Monoclonal	SP142	Spring Biosciences	ab228462	1:100, incubate for 30 min at room temperature
B7x	Monoclonal	1H3	[31]	N/A	1:500, incubate overnight at 4 °C
HHLA2	Monoclonal	566.1	[15,32]	N/A	1:500, incubate overnight at 4 °C
B7-H3	Polyclonal	N/A	R&D sciences [33]	BAF1027	5 μg/mL, incubate overnight at 4 °C

**Table 2:** Primer sequences for quantitative PCR.

Gene Name	Direction	Primer Sequence
*CD274*	forward	5′-TGGCATTTGCTGAACGCATTT-3′
	reverse	5′-TGCAGCCAGGTCTAATTGTTTT-3′
*CD276*	Forward	5′-CTGGCTTTCGTGTGCTGGAGAA-3′
	reverse	5′-GCTGTCAGAGTGTTTCAGAGGC-3′
*VTCN1*	forward	5′-TCTGGGCATCCCAAGTTGAC-3′
	reverse	5′-TCCGCCTTTTGATCTCCGATT-3′
*HHLA2*	forward	5′-TACAAAGGCAGTGACCATTTGG-3′
	reverse	5′-AGGTGTAAATTCCTTCGTCCAGA-3′
*GAPDH*	forward	5′-GGAGCGAGATCCCTCCAAAAT-3′
	reverse	5′-GGCTGTTGTCATACTTCTCATGG-3′

**Table 3:** Primary antibodies for Western blot.

Protein	Company	Catalog No.	Source	Concentration
MET	Cell Signaling Technology	8198	Rabbit	1:1000
phospho-MET	Cell Signaling Technology	3077	Rabbit	1:1000
AKT	Cell Signaling Technology	9272	Rabbit	1:1000
phospho-AKT	Cell Signaling Technology	9271	Rabbit	1:1000
P42/44 MAPK	Cell Signaling Technology	9102	Rabbit	1:1000
phospho-p42/44 MAPK	Cell Signaling Technology	9101	Rabbit	1:1000
GAPDH	Cell Signaling Technology	5174	Rabbit	1:1000

**Table 4:** Patient characteristics by expression of immune checkpoints.

	PD-L1			B7X			B7H3			HHLA2		
Parameter	Negative	Positive	*p*	Negative	Positive	*p*	Negative	Positive	*p*	Negative	Positive	*p*
Age, year	70	72	0.602	68	72	0.277	69.5	71.33	0.664	74.3	70.5	0.579
Gender			0.470			0.482			0.288			0.580
Female	4 (44.4%)	16 (59.3%)		5 (41.7%)	15 (60%)		7 (70.0%)	13 (48.1%)		1 (33.3%)	19 (55.9%)	
Male	5 (55.6%)	11 (40.7%)		7 (58.3%)	10 (40%)		3 (30.0%)	14 (51.9%)		2 (66.7%)	15 (44.1%)	
Smoking status			0.921			0.207			0.282			0.536
Never smoker	1 (12.5%)	2 (8.0%)		1 (9.1%)	2 (8.7%)		0 (0.0%)	3 (11.5%)		0 (0.0%)	3 (9.7%)	
Former smoker	5 (62.5%)	17 (68.0%)		5 (45.5%)	17 (73.9%)		7 (87.5%)	15 (57.7%)		2 (66.7%)	20 (64.5%)	
Active smoker	2 (25.0%)	6 (24.0%)		5 (0.0%)	4 (0.0%)		1 (12.5%)	8 (30.8%)		1 (33.3%)	8 (25.8%)	
Stage			0.823			0.920			0.541			0.309
I	4 (44.4%)	8 (29.6%)		4 (33.3%)	8 (32.0%)		5 (50.0%)	7 (25.9%)		0 (0.0%)	12 (35.3%)	
II	3 (33.3%)	11 (40.7%)		5 (41.7%)	10 (40.0%)		3 (30.0%)	12 (44.4%)		1 (33.3%)	14 (41.2%)	
III	2 (22.2%)	7 (25.9%)		3 (25.0%)	6 (24.0%)		2 (20.0%)	7 (25.9%)		2 (66.7%)	7 (20.6%)	
IV	0 (0.0%)	1 (3.7%)		0 (0.0%)	1 (4.0%)		0 (0.0%)	1 (3.7%)		0 (0.0%)	1 (2.9%)	
Lymph node			0.427			0.478			1.000			1.000
Negative	7 (77.8%)	14 (56.0%)		8 (72.7%)	14 (58.3%)		6 (66.7%)	16 (61.5%)		2 (66.7%)	20 (62.5%)	
Positive	2 (22.2%)	11 (44.0%)		3 (27.3%)	10 (41.7%)		3 (33.3%)	10 (38.5%)		1 (33.3%)	12 (37.5%)	
Mutation status												
*MET* mutation			0.160			1.000			1.000			1.000
Negative	9 (100.0%)	19 (70.4%)		10 (83.3%)	19 (17.0%)		8 (80.0%)	21 (77.8%)		3 (100.0%)	26 (76.5%)	
Positive	0 (0.0%)	8 (29.6%)		2 (16.7%)	6 (24.0%)		2 (20.0%)	6 (22.2%)		0 (0.0%)	8 (23.5%)	
*KRAS* mutation			0.652			1.000			0.655			0.431
Negative	8 (88.9%)	21 (80.8%)		10 (83.3%)	20 (83.3%)		9 (90.0%)	21 (80.8%)		2 (66.7%)	28 (84.8%)	
Positive	1 (11.1%)	5 (19.2%)		2 (16.7%)	4 (16.7%)		1 (10.0%)	5 (19.2%)		1 (33.3%)	5 (15.2%)	
TIL score			0.223			1.000			1.000			0.536
Low (<30%)	8 (88.9%)	17 (65.4%)		8 (66.7%)	17 (73.9%)		7 (70.0%)	18 (72.0%)		2 (100.0%)	23 (69.7%)	
High (≥30%)	1 (11.1%)	9 (34.6%)		4 (33.3%)	6 (26.1%)		3 (30.0%)	7 (28.0%)		0 (0.0%)	10 (30.3%)	
B7X			1.000									
Negative	3 (33.3%)	9 (33.3%)										
Positive	6 (66.7%)	18 (66.7%)										
B7H3			0.226									
Negative	4 (44.4%)	6 (22.2%)										
Positive	5 (55.6%)	21 (77.8%)										
HHLA2			1.000									
Negative	0 (0.0%)	2 (7.4%)										
Positive	9 (100.0%)	25 (92.6%)										

**Table 5:** Expression of immune checkpoints in epithelial and sarcomatoid components. A. Differential expression of immune checkpoints. B. PD-L1 co-expression with other immune checkpoints on epithelial and sarcomatoid components. C. Alternative immune checkpoint expression in dual PD-L1-negative tumors.

A.								
	PD-L1		B7x		B7H3		HHLA2	
	Median (interquartile range)	*p*	Median (interquartile range)	*p*	Median (interquartile range)	*p*	Median (interquartile range)	*p*
Percentage of tumor		0.010		0.008		0.053		0.553
Epithelial	50 (0–75)		25 (0–75)		0 (0–50)		50 (75–100)	
Sarcomatoid	0 (0–58.5)		0 (0–23.75)		50 (0–75)		95 (57.5–95.0)	
H-Score		0.014		0.013		0.176		0.9
Epithelial	75 (0–150)		25 (0–75)		0 (0–100)		100 (75–180)	
Sarcomatoid	0 (0–100)		0 (0–23.75)		75 (0–150)		100 (0–160)	
B.								
	Any Other Immune Checkpoint Expression							
	Epithelial				Sarcomatoid			
PD-L1 Epithelial	Negative	Positive		PD-L1 Sarcomatoid	Negative	Positive		
Negative	2 (7.1%)	13 (46.4%)		Negative	0 (0.0%)	8 (26.7%)		
Positive	0 (0.0%)	13 (46.4%)		Positive	1 (3.3%)	21 (70.0%)		
C.								
		Epithelial			Sarcomatoid			
B7x		4 (50.0%)			3 (33.3%)			
B7H3		3 (37.5%)			3 (33.3%)			
HHLA2		8 (100%)			6 (66.7%)			

## Data Availability

The supporting data are not publicly available due to research participant privacy restrictions.

## Supplementary Materials

The following supporting information can be downloaded at: https://www.mdpi.com/article/10.3390/cancers15133372/s1: Table S1. Immune checkpoint expression by genetic alterations and histologic subcomponents; Table S2. Association of tumor-infiltrating lymphocytes with genetic alterations; Table S3. Full Cox regression model for survival analysis; File S1: Uncropped Western blot images.
