# Supplementary material for: Heterogeneous Expression of PD-L1, B7x, B7-H3, and HHLA2 in Pulmonary Sarcomatoid Carcinoma and the Related Regulatory Signaling Pathways
Source: Cancers (Basel). 2023 Jun 27;15(13):3372. doi: 10.3390/cancers15133372 (PMC10340395; doi:10.3390/cancers15133372)
Supplement: Supplementary file 1 [file cancers-15-03372-s001.zip › File S1. psc-original WB images with molecular weights.pptx]

## Slide 1
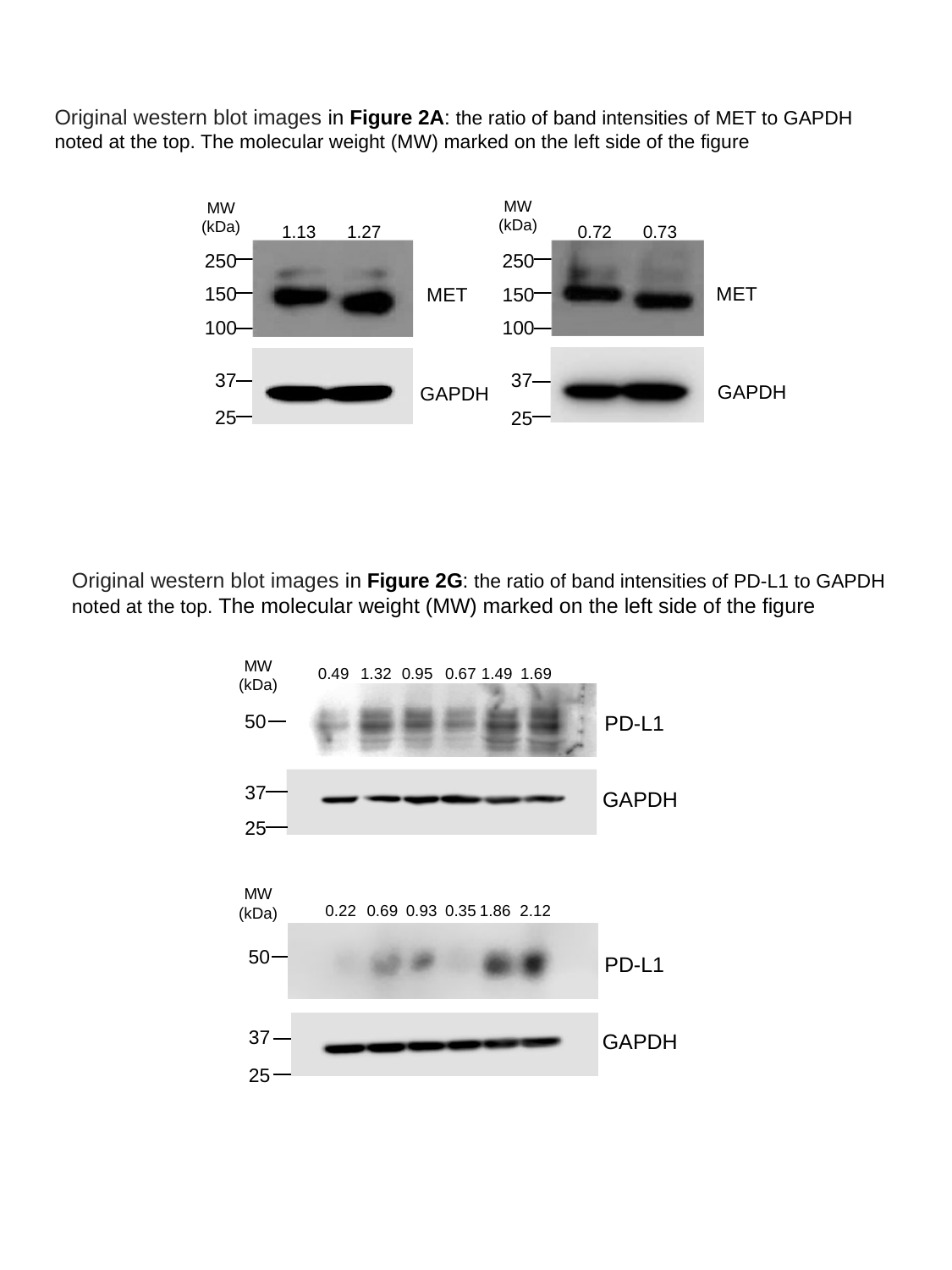

Original western blot images in Figure 2A: the ratio of band intensities of MET to GAPDH noted at the top. The molecular weight (MW) marked on the left side of the figure
MW
(kDa)
MW
(kDa)
1.13
1.27
0.72
0.73
250
250
MET
MET
150
150
100
100
37
37
GAPDH
GAPDH
25
25
Original western blot images in Figure 2G: the ratio of band intensities of PD-L1 to GAPDH noted at the top. The molecular weight (MW) marked on the left side of the figure
MW
(kDa)
0.49
1.32
0.95
0.67
1.49
1.69
E
50
PD-L1
37
GAPDH
25
MW
(kDa)
0.22
0.69
0.93
0.35
1.86
2.12
50
PD-L1
37
GAPDH
25

## Slide 2
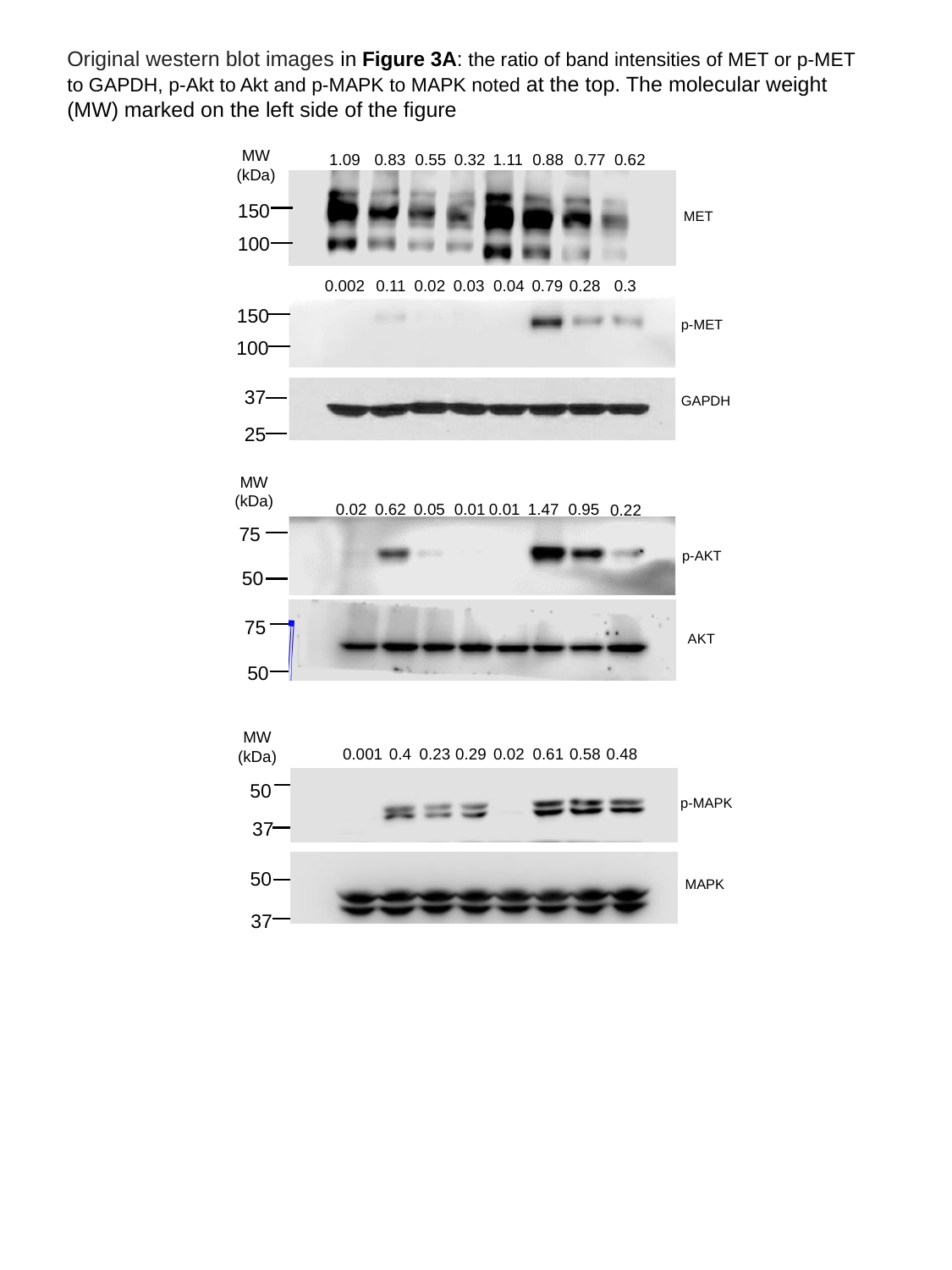

Original western blot images in Figure 3A: the ratio of band intensities of MET or p-MET to GAPDH, p-Akt to Akt and p-MAPK to MAPK noted at the top. The molecular weight (MW) marked on the left side of the figure
MW
(kDa)
1.09
0.83
0.55
0.32
1.11
0.88
0.77
0.62
150
MET
100
0.002
0.11
0.02
0.03
0.04
0.79
0.28
0.3
150
p-MET
100
37
GAPDH
25
MW
(kDa)
0.02
0.62
0.05
0.01
0.01
1.47
0.95
0.22
75
p-AKT
50
75
AKT
50
MW
(kDa)
0.001
0.4
0.23
0.29
0.02
0.61
0.58
0.48
50
p-MAPK
37
50
MAPK
37

## Slide 3
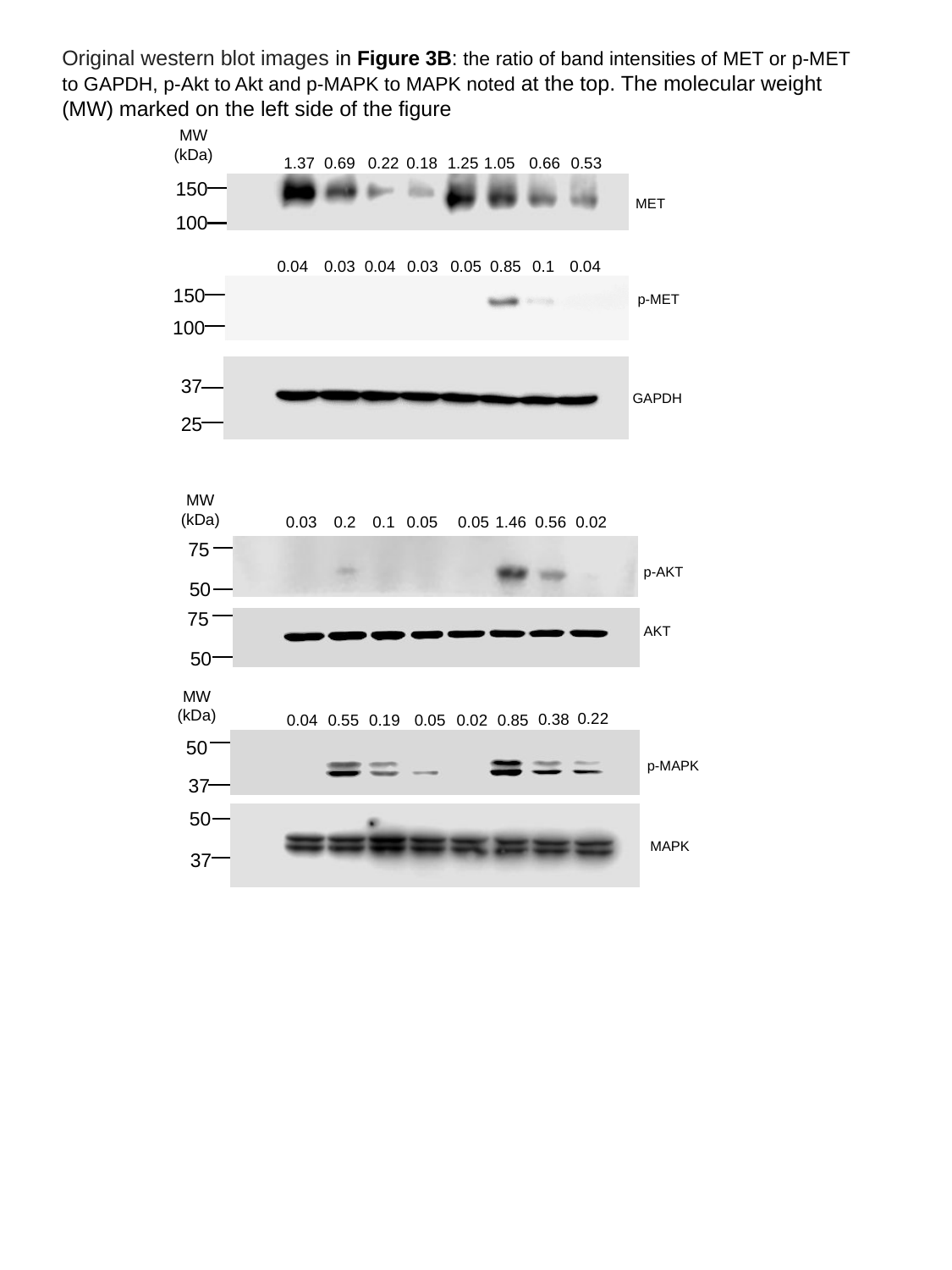

Original western blot images in Figure 3B: the ratio of band intensities of MET or p-MET to GAPDH, p-Akt to Akt and p-MAPK to MAPK noted at the top. The molecular weight (MW) marked on the left side of the figure
MW
(kDa)
1.37
0.69
0.22
0.18
1.25
1.05
0.66
0.53
150
MET
100
0.04
0.03
0.04
0.03
0.05
0.85
0.1
0.04
150
p-MET
100
37
GAPDH
25
MW
(kDa)
0.03
0.2
0.1
0.05
0.05
1.46
0.56
0.02
75
AKT
p-AKT
50
75
AKT
50
MW
(kDa)
0.22
0.38
0.55
0.05
0.02
0.85
0.04
0.19
50
p-MAPK
37
50
MAPK
37

## Slide 4
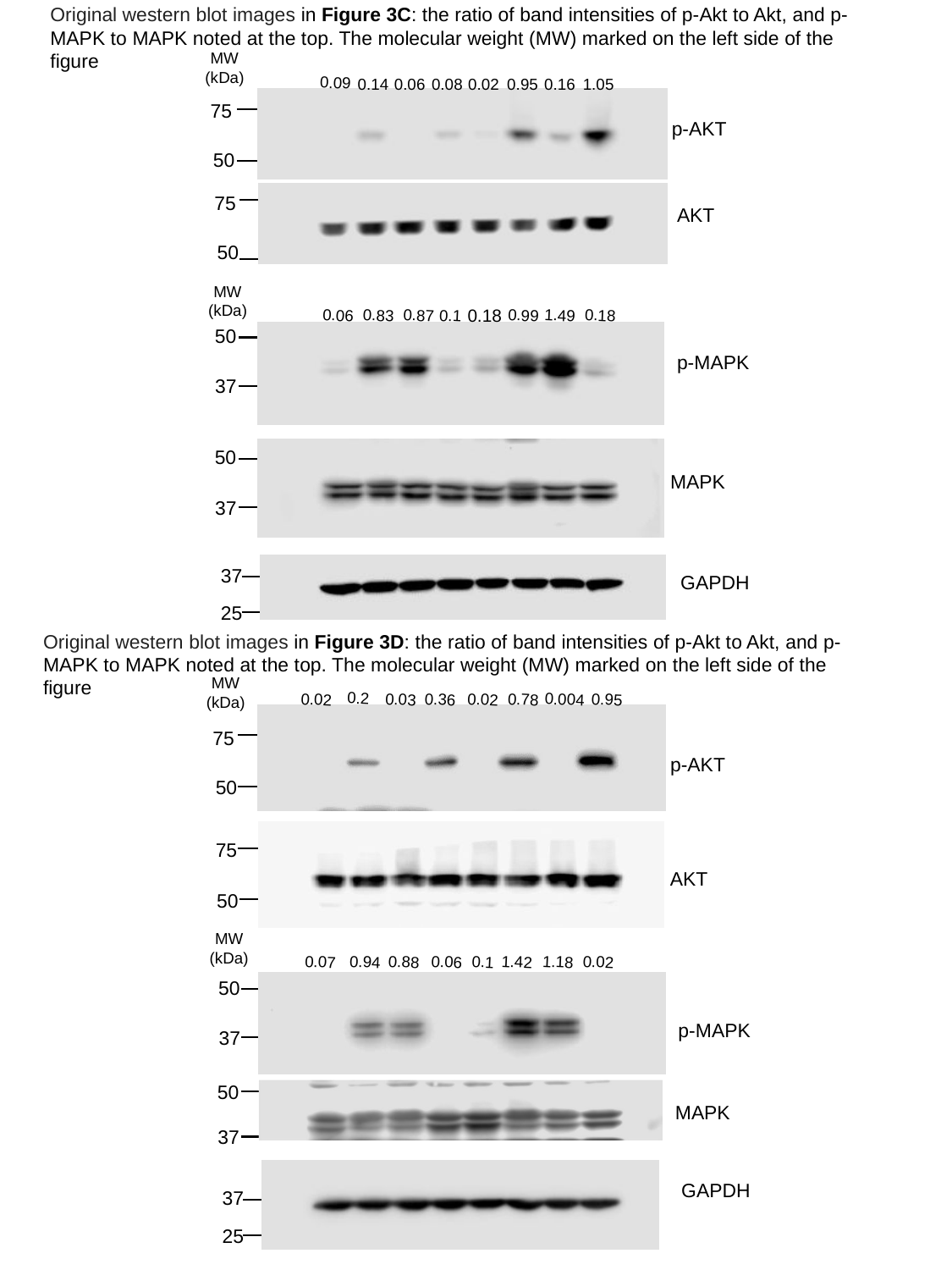

Original western blot images in Figure 3C: the ratio of band intensities of p-Akt to Akt, and p-MAPK to MAPK noted at the top. The molecular weight (MW) marked on the left side of the figure
MW
(kDa)
0.09
0.14
0.06
0.08
0.02
0.95
0.16
1.05
75
p-AKT
50
75
AKT
50
MW
(kDa)
0.18
0.06
0.83
0.87
0.99
1.49
0.18
0.1
50
p-MAPK
37
50
MAPK
37
37
GAPDH
25
Original western blot images in Figure 3D: the ratio of band intensities of p-Akt to Akt, and p-MAPK to MAPK noted at the top. The molecular weight (MW) marked on the left side of the figure
MW
(kDa)
0.2
0.004
0.02
0.03
0.36
0.02
0.78
0.95
75
p-AKT
50
75
AKT
50
MW
(kDa)
0.07
0.94
0.88
0.06
1.42
1.18
0.02
0.1
50
p-MAPK
37
50
MAPK
37
GAPDH
37
25

## Slide 5
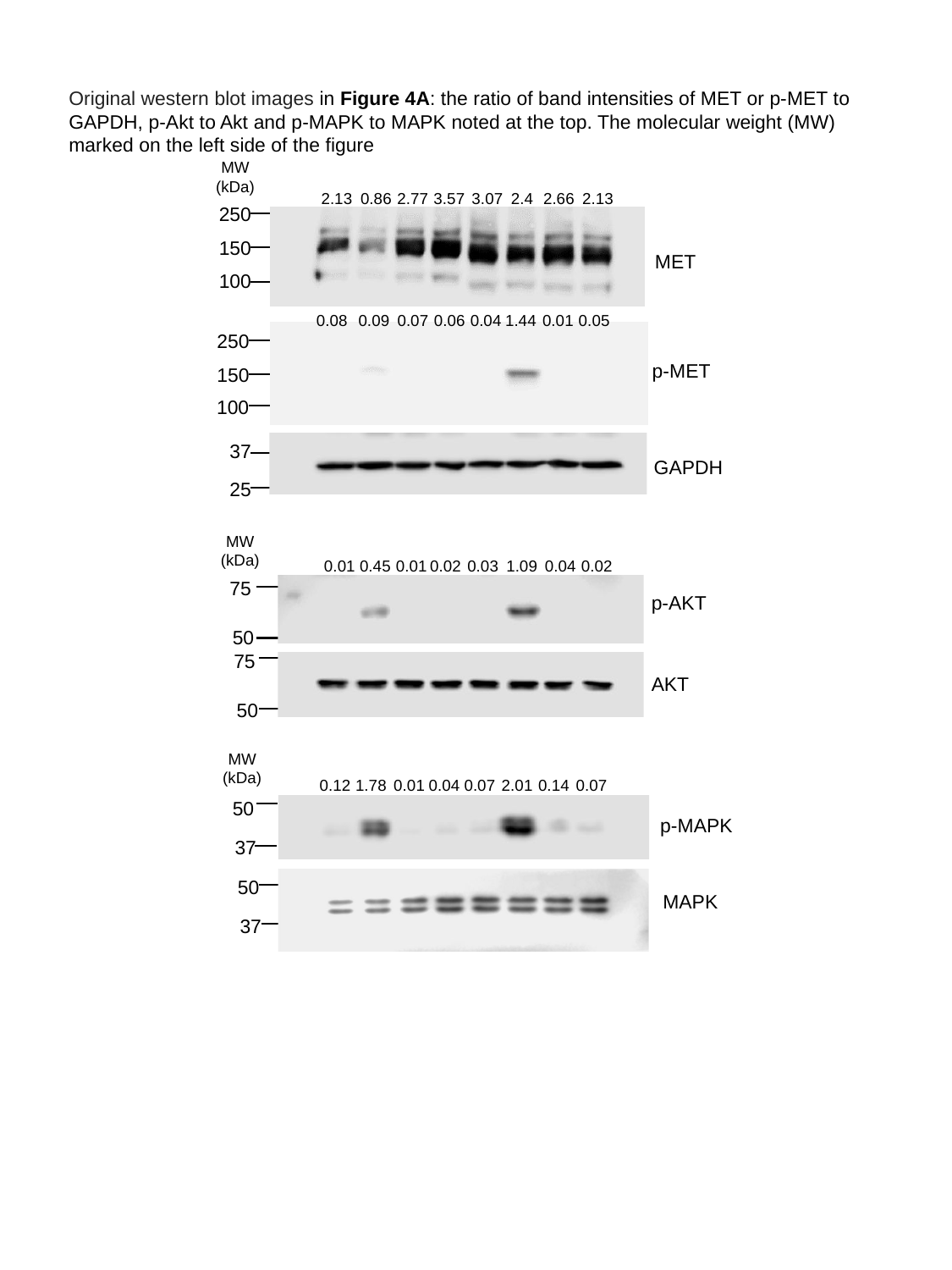

Original western blot images in Figure 4A: the ratio of band intensities of MET or p-MET to GAPDH, p-Akt to Akt and p-MAPK to MAPK noted at the top. The molecular weight (MW) marked on the left side of the figure
MW
(kDa)
2.13
0.86
2.77
3.57
3.07
2.4
2.66
2.13
250
150
MET
100
0.08
0.09
0.07
0.06
0.04
1.44
0.01
0.05
250
p-MET
150
100
37
GAPDH
25
MW
(kDa)
0.01
0.45
0.01
0.02
0.03
1.09
0.04
0.02
75
p-AKT
50
75
AKT
50
MW
(kDa)
0.12
1.78
0.01
0.04
0.07
2.01
0.14
0.07
50
p-MAPK
37
50
MAPK
37

## Slide 6
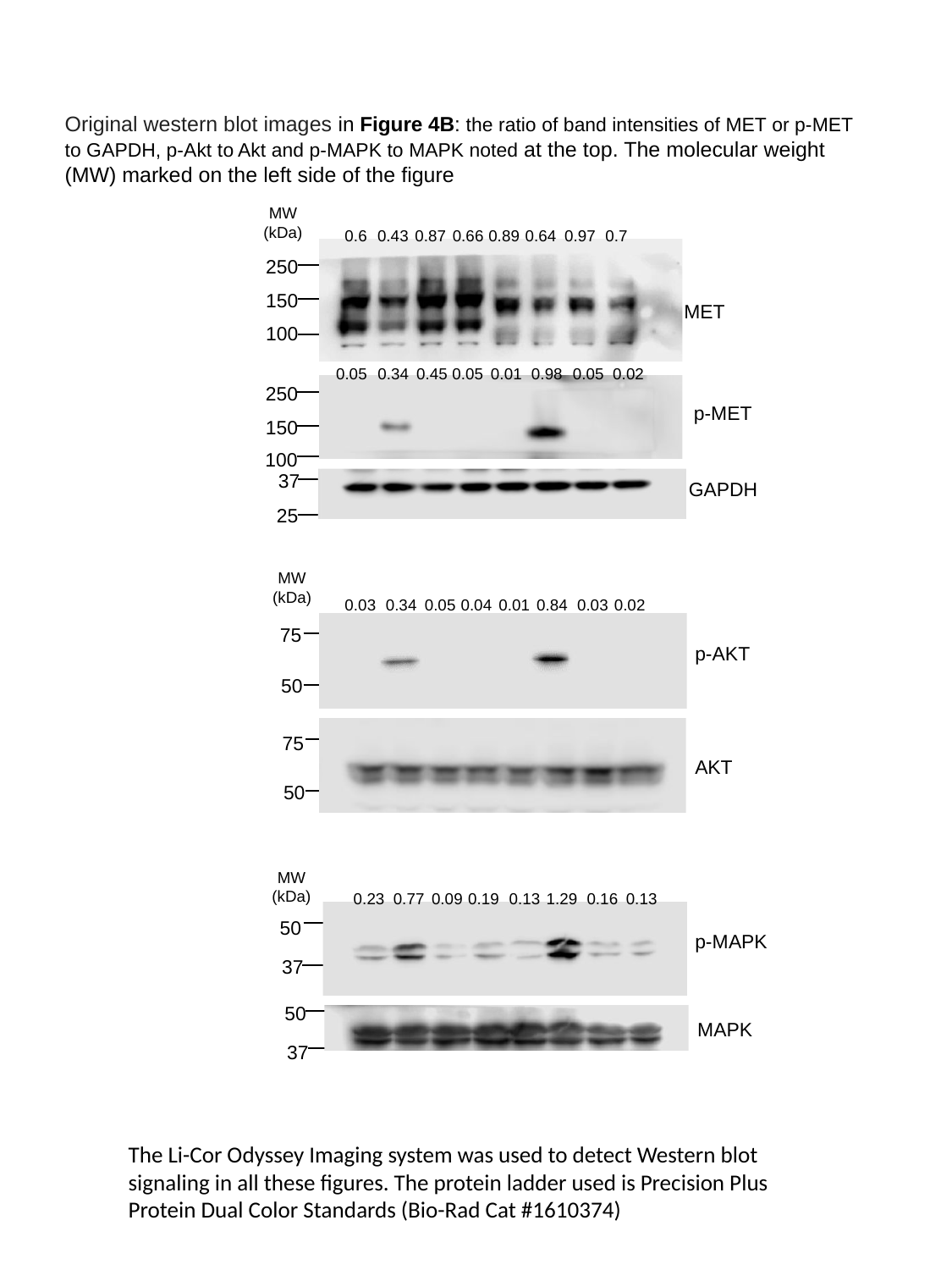

Original western blot images in Figure 4B: the ratio of band intensities of MET or p-MET to GAPDH, p-Akt to Akt and p-MAPK to MAPK noted at the top. The molecular weight (MW) marked on the left side of the figure
MW
(kDa)
0.6
0.43
0.87
0.66
0.89
0.64
0.97
0.7
250
150
MET
100
0.05
0.34
0.45
0.05
0.01
0.98
0.05
0.02
250
p-MET
150
100
37
GAPDH
25
MW
(kDa)
0.02
0.03
0.34
0.05
0.04
0.01
0.84
0.03
75
p-AKT
50
75
AKT
50
MW
(kDa)
0.23
0.77
0.09
0.19
0.13
1.29
0.16
0.13
50
p-MAPK
37
50
MAPK
37
The Li-Cor Odyssey Imaging system was used to detect Western blot signaling in all these figures. The protein ladder used is Precision Plus Protein Dual Color Standards (Bio-Rad Cat #1610374)
