# Supplementary material for: Heterogeneous Expression of PD-L1, B7x, B7-H3, and HHLA2 in Pulmonary Sarcomatoid Carcinoma and the Related Regulatory Signaling Pathways
Source: Cancers (Basel). 2023 Jun 27;15(13):3372. doi: 10.3390/cancers15133372 (PMC10340395; doi:10.3390/cancers15133372)
Supplement: Supplementary file 1 [file cancers-15-03372-s001.zip › Supplementary Tables.pdf]

Supplementary Table S1. Genetic Mutations and Immune Checkpoints

	PD-L1		B7x		B7H3		HHLA2	
	Median (interquartile range)	P	Median (interquartile range)	P	Median (interquartile range)	P	Median (interquartile range)	P
MET Exon 14 Skipping								
Epithelial (%)		0.540		0.834		0.947		0.051
Mutated	56 (4-100)		28 (0-69)		0 (0-69)		100 (93-100)	
Wildtype	50 (0-75)		18 (0-76)		0 (0-50)		80 (25-100)	
Epithelial (H-Score)		0.647		0.919		0.982		**<0.001**
Mutated	75 (4-200)		28 (0-69)		0 (0-125)		200 (185-200)	
Wildtype	75 (0-75)		10 (0-75)		0 (0-100)		100 (50-100)	
Sarcomatoid (%)		0.079		0.718		0.067		0.242
Mutated	56 (0-100)		0 (0-20)		75 (50-85)		100 (80-100)	
Wildtype	0 (0-35)		0 (0-25)		50 (0-75)		80 (15-100)	
Sarcomatoid (H-Score)		0.198		0.718		**0.017**		**0.031**
Mutated	88 (0-138)		0 (0-20)		150 (88-170)		200 (50-200)	
Wildtype	0 (0-55)		0 (0-25)		50 (0-94)		100 (0-115)	
KRAS								
Epithelial (%)		0.628		0.483		0.154		**0.026**
Mutated	30 (8-60)		50 (0-85)		0 (0-6)		50 (8 - 81)	
Wildtype	75 (0-88)		10 (0-75)		0 (0-50)		100 (80-100)	
Epithelial (H-Score)		0.712		0.438		0.155		**0.041**
Mutated	30 (15-80)		50 (0-85)		0 (0-6)		63 (8-113)	
Wildtype	75 (0-200)		10 (0-75)		0 (0-100)		100 (85-200)	
Sarcomatoid (%)		0.852		0.503		0.309		0.116
Mutated	15 (0-55)		0 (0-63)		75 (38-78)		75 (0-88)	
Wildtype	0 (0-0)		0 (0-20)		50 (0-75)		100 (64-100)	
Sarcomatoid (H-Score)		0.894		0.503		0.426		0.150
Mutated	15 (0-110)		0 (0-63)		75 (38-155)		38 (0-113)	
Wildtype	0 (0-0)		0 (0-20)		62.5 (0-150)		100 (100-175)	

Supplementary Table S2. Association of Tumor Infiltrating Lymphocyte with Genetic Mutations

	Tumor Infiltrating Lymphocyte Percentage		p
	<30%	≥ 30%	
MET Exon 14 skipping			0.027
Negative	22 (88.0%)	5 (50.0%)	
Positive	3 (12.0%)	5 (50.0%)	
KRAS Mutation			1.000
Negative	21 (84.0%)	8 (88.9%)	
Positive	4 (16.0%)	1(11.1%)	

All p values are from chi-square test.

Supplementary Table S3. Full Cox Regression Model for Survival

Factors	Hazard Ratio (95% CI)	p
Male vs. female	0.769 (0.089 - 6.676)	0.812
Age ≥ 65 vs. < 65	1.815 (0.143 - 23.013)	0.646
Sarcomatoid proportion (continuous)	1.043 (1.007 - 1.080)	**0.018**
Stage		
II vs. I	5.090 (0.622 - 41.676)	0.129
III vs. I	1.496 (0.215 - 10.411)	0.684
IV vs. I	41.302 (0.509 - 3350.704)	0.097
PDL1 positive vs. negative	2.245 (0.304 - 16.56)	0.428
B7x positive vs. negative	0.724 (0.140 - 3.756)	0.701
B7H3 positive vs. negative	0.611 (0.077 - 4.87)	0.641
HHLA2 positive vs. negative	1.522 (0.036 - 64.877)	0.826
MET exon 14 skipping positive vs. negative	0.727 (0.112 - 4.738)	0.739
KRAS mutation positive vs. negative	1.260 (0.13 - 12.246)	0.842
